# Genome-based therapeutic interventions for β-type hemoglobinopathies

**DOI:** 10.1186/s40246-021-00329-0

**Published:** 2021-06-05

**Authors:** Kariofyllis Karamperis, Maria T. Tsoumpeli, Fotios Kounelis, Maria Koromina, Christina Mitropoulou, Catia Moutinho, George P. Patrinos

**Affiliations:** 1 Department of Pharmacy, School of Health Sciences, Laboratory of Pharmacogenomics and Individualized Therapy, University of Patras, Patras, Greece; 2 The Golden Helix Foundation, London, UK; 3 School of Veterinary Medicine and Science, University of Nottingham, Nottingham, UK; 4 Department of Computing, Group of Large-Scale Data & Systems, Imperial College London, London, UK; 5 Garvan-Weizmann Centre for Cellular Genomics, Garvan Institute of Medical Research, Darlinghurst, Sydney, Australia; 6 College of Medicine and Health Sciences, Department of Pathology, United Arab Emirates University, Al-Ain, United Arab Emirates; 7 Zayed Center of Health Sciences, United Arab Emirates University, Al-Ain, United Arab Emirates

**Keywords:** β-thalassemia, Sickle cell disease, Pharmacogenomics, Gene addition, Genome editing technologies, Gene therapy, Viral and non-viral vectors

## Abstract

For decades, various strategies have been proposed to solve the enigma of hemoglobinopathies, especially severe cases. However, most of them seem to be lagging in terms of effectiveness and safety. So far, the most prevalent and promising treatment options for patients with β-types hemoglobinopathies, among others, predominantly include drug treatment and gene therapy. Despite the significant improvements of such interventions to the patient’s quality of life, a variable response has been demonstrated among different groups of patients and populations. This is essentially due to the complexity of the disease and other genetic factors. In recent years, a more in-depth understanding of the molecular basis of the β-type hemoglobinopathies has led to significant upgrades to the current technologies, as well as the addition of new ones attempting to elucidate these barriers. Therefore, the purpose of this article is to shed light on pharmacogenomics, gene addition, and genome editing technologies, and consequently, their potential use as direct and indirect genome-based interventions, in different strategies, referring to drug and gene therapy. Furthermore, all the latest progress, updates, and scientific achievements for patients with β-type hemoglobinopathies will be described in detail.

## Background

Existing treatments in patients with β-type hemoglobinopathies are usually lacking therapeutic efficacy and therefore the research interest has focused into different directions and strategies. Nowadays, the development of current and new technologies has led to the direct and indirect integration of genome-based therapeutic interventions to both drug and gene therapy, respectively. In the first case, pharmacogenomics, acting as genome-guided treatment, can potentially differentiate the patients to hydroxyurea response and hence ameliorate the disease severity. In gene therapy, the upgrade of intervention tools along with gene addition and genome editing technologies has significantly contributed to the optimization of the whole process, thus demonstrating a series of benefits in clinical trials. In this review, we provide a comprehensive overview of the most emerging technologies for drug and gene therapy, and moreover, we attempt to link the gaps between the identification of the disease to the selection of approach strategy, including all the strengths and weaknesses.

## Introduction

Hemoglobinopathies are the most common monogenic disease and one of the most devastating health diseases around the world. Approximately, 2–7% are carriers of inherited hemoglobin disorders by either quantitative or/and qualitative abnormalities of the hemoglobin (Hb) molecule [1, 2]. Among them, β-thalassemia and sickle cell disease (SCD), also referred to as β-type hemoglobinopathies, are the most prevalent and with the greatest public health impact in terms of expenditure [3]. Sickle cell disease results from a single nucleotide substitution (SNP, rs334) in the β-globin gene (*HBB)* that causes the formation of an unstable sickling form of hemoglobin called HbS, whereas β-thalassemias can be caused by diverse mutations (single nucleotide substitution, deletions, insertions, etc.) in the *HBB* gene, respectively [4]. On a worldwide scale, hemoglobinopathies are distributed at high frequencies in the Mediterranean region, Middle East, Southeast Asia, the Indian subcontinent, and Sub-Saharan Africa [5, 6]. In general, β-type hemoglobinopathies are common in Mediterranean regions, South and Central America, Saudi Arabia, sub-Saharan Africa, Indian sub-continent. In the last few years, carriers with β-types of hemoglobinopathies have significantly increased and new cases have spread to the surrounding areas, such as the Caribbean islands, and part of North America due to migration [7–10]. Approximately, 300,000 to 400,000 newborns are detected with a serious hemoglobin disorder, per annum and the vast majority, up to 90%, are derived from low or middle-income countries [11, 12]. Based on the above, the gradual increase of the incidence rates alongside the current geographical distribution of the β-type hemoglobinopathies leading to its serious widespread. Curative approaches and strategies to both prevention and treatment are considered highly important as the “burden of the disease” is maintained at high-risk levels [13].

### Current therapeutic approaches

The standard treatment selection is based on the identification and subsequent molecular characterization of patients with inherited blood disorders is a prerequisite for the selection of appropriate treatment. In addition, other parameters including clinical manifestation (may range from none to severe) and severity grade are considered important for the stratification of patients [14, 15]. Therefore, clinical severity is highly variable, and the curative approach is defined based on all the above parameters. Generally, in mild forms, with non-clinical significance and minor symptoms, supportive medications are usually given to prevent or to avoid possible deterioration of the disease. In contrast, patients with major forms of hemoglobin disorders usually experience severe anemia, pain, fatigue, etc., and thus these conditions can be life-threatening. Into this group, severe sickle cell disease (HbSS) and β-thalassemia major or Cooley’s anemia (β^0^/β^0^) are characterized as the most severe forms (also known as major β-types) with considerable morbidity and mortality [16]. Treatment options in patients with β-thalassemia include drug treatment to increase levels of fetal hemoglobin, iron chelation therapy, splenectomy, antioxidants, and regular blood transfusions, whereas, in severe cases of patients with sickle cell disease, drug treatment with hydroxyurea as well as regular blood transfusions are listed as first-line options [17, 18]. It should be highlighted that all the above treatment options are largely supportive and therefore combination therapies are usually recommended, although with an increased risk of side effects [6]. Specifically, the addition of iron chelation therapy is a common phenomenon as lifelong dependence on red blood cell transfusions might lead to several complications such as iron overload and splenomegaly [19].

To date, hematopoietic stem cell transplantation (HSCT) and more precisely, allogeneic stem cell transplantation is the only available curative treatment. Significant advances in medical management and transplant-related complications have led to a more frequent implementation to those patients that are considered eligible. Unfortunately, the percentage of patients with high success rates does not exceed 10% and hence accessibility remains limited. The most common limitations are the lack of suitable donors and the high probability of immune side effects. Human leukocyte antigens (HLA) matching between donors and recipients largely defines whether a transplant can be performed with a high probability of success [18, 20–22]. Other factors, such as the patient’s clinical condition (comorbidity) and age, are also taken into account as additional limitations and therefore success rates may vary greatly among patients. The above knowledge together with the complexity of β-type hemoglobinopathies patients and the limited curative options indicate that a tailored intervention based on the genetic constitution of each patient would be beneficial [23, 24]. The striking improvement of genome technology allows us to explore different novel strategies, both pharmacological and genetic, aiming to substantially improve treatment or at least, to ameliorate clinical manifestations to a significant level. Following these two different strategies, the research interest has focused either on the development of autologous stem cell gene-based therapy by using newly developed technologies or on the improvement of drug efficacy with hydroxyurea, utilizing pharmacogenomics technologies [22, 25]. All the recent progress and how genome-based therapeutic technologies serve their future implementation are described below.

### Drug treatment—modulation of fetal hemoglobin

The normal developmental transition from fetal (α2γ2; HbF) to adult hemoglobin production (α2β2; HbA) is an area of long-term interest in the context of therapeutic approaches. It is common knowledge that maintaining high levels of HbF can ameliorate the clinical complications of SCD to a lesser extent extent of β-thalassemia, due to its molecular structure and abilities (binds to oxygen with more affinity and disrupts sickle hemoglobin polymerization) [13, 26]. Despite the concerted efforts to identify an effective way to re-activate the production of HbF in therapeutic levels, there has been limited success following severe adverse events [27, 28]. Up to date, a variety of pharmacological agents, following different cellular, epigenetic, and molecular mechanisms of action have been designed and developed [8]. The most common are deoxyribonucleic acid (DNA) methyltransferase inhibitors (5-azacytidine, decitabine), histone deacetylases inhibitors, histone deacetylase inhibitors (HDACi) (trichostatin A, apicidin, sodium butyrate), monoamine oxidase inhibitors (tranylcypromine), mammalian target of rapamycin inhibitor, mechanistic target of rapamycin (mTOR) (sirolimus), and ribonucleotide reductase inhibitors such as hydroxyurea. Lately, immunomodulatory drugs, IMiDs (pomalidomide, thalidomide) are strongly recommended, as they can improve the β-globin imbalance [15, 29–35]. From all the above, hydroxyurea is the most commonly used drug for the treatment of SCD and the only approved until 2019, for clinical use from the European Medicines Agency (EMA 2007) [36] and the US Food and Drug Administration (FDA 2017) [37]. Currently, new drugs like voxelotor (also known as GBT440) and luspatercept, both approved by FDA and EMA, have been released for the treatment of SCD and β-thalassemia, respectively [38–41]. There is a great expectation regarding the use of voxelotor as “it consists the first of its kind,” meaning that it could potentially alter the underlying SCD pathology. After a series of clinical trials (NCT02285088, NCT03041909), this drug has shown great benefits by increasing the affinity between Hb and oxygen resulting in the inhibition of drepanocytosis of the RBCs and therefore, to a significant improvement in patients’ quality of life [42, 43]. Subsequently, in phase 3 clinical trial called GBT_HOPE (NCT03036813), voxelotor established efficacy and safety even with concomitant administration of placebo (hydroxyurea), in patients with SCD (*N* = 274). Based on the outcome measures, the hypothesis that these drugs may have complementary mechanisms is further strengthened, as hemolysis decreased and, conversely, hemoglobin concentration increased significantly [44, 45]. The drug luspatercept can be characterized as a valuable adjunct for ameliorating anemia conditions especially, in those patients with severe forms of β-thalassemia [46].

It should be noted that most of the mentioned drugs are used for sickle cell disease, however, due to their ability to increase fetal hemoglobin and hence to improve clinical and hematological abnormalities, could also be effective in β-thalassemias [47–49]. Hydroxyurea, due to its long-term use, has been extensively studied for the treatment of β-type hemoglobinopathies and the results so far confirm a wide variation in drug response, among individuals. Depending on HbF levels after administration of hydroxyurea, a patient is classified as a hydroxyurea (HU) responder (HU) or a HU non-responder (partial responders are also characterized as non-responders). These differences in HU response derive from the fact that complex molecular mechanisms and genetic factors underlying hemoglobin switching affect the synthesis of HbF. Nevertheless, multiple genetic studies have successfully characterized key variants, mostly in regulatory pathways where are associated with HbF induction and likely to HU treatment [27, 50–53].

## Genome-guided treatment: pharmacogenomics for β-type hemoglobinopathies

Personalized medicine and in particular, pharmacogenomics is an emerging research field aiming to analyze genomic profiles and consequently identify associations of genomic variants with drug response. As an emerging field, it has already shown its application for a variety of diseases, including inherited blood disorders, and it could potentially be applied in different ways for β-type hemoglobinopathies, both in prevention and treatment. More precisely, an estimate of 25% of patients with β-thalassemia or sickle-cell disease has been characterized as poor metabolizers or non-responders to HU treatment [25, 54]. Pharmacogenomics could be beneficial toward delineating the genetic factors in which the augmenting of HbF levels may vary considerably among β-thalassemia and SCD. Taken from this, drug treatment can be tailored to those individuals that appear to respond to a particular drug while avoiding any potential adverse drug reactions to non-responders, based on the genetic makeup. It becomes clear then that the concept of implementing pharmacogenomics as a genome-guided treatment for β-type hemoglobinopathies is of great interest.

### Drug therapy: updates for hydroxyurea response

Pharmacogenomics researchers may use a combination of high-throughput sequencing technologies and disease or drug-related databases, toward identifying and detecting biomarkers for β-type hemoglobinopathies. As indirect genome-based therapeutic intervention has the potential to predict the outcome of a drug intervention, and in particular to hydroxyurea, delivering personalized and targeted drug therapy [55, 56]. Even though this approach is still at the early phase of development concerning hemoglobinopathies, significant SNPs in genes localized within as well outside the human β-globin gene locus have been detected with an important role in HbF augmenting and moreover to HU treatment. In fact, genome-wide association studies (GWAS) have revealed a strong association between the quantitative trait loci namely Xmnl-HBG2, HBS1L–MYB, and B cell lymphoma/leukemia 11A (BCL11A) with the range of HbF induction [57–61]. These findings broadened our knowledge for a better understanding of the baseline HbF and therefore defined as starting points for a series of new targets. Based on the latest in vitro and in silico findings, the degree of HbF induction to treatment with HU appears to be an inherited trait and thus *cis* and *trans*-regulatory elements or quantitative trait loci are considered crucial factors, for drug treatment and secondary to disease severity [62–67].

In particular, Ma and co-workers (2007) studied the association of specific tag-SNPs (*N* = 320) with HU treatment, in patients with sickle cell disease (*N* = 137), with African American origin with HU treatment. In total, a significant association was observed in 17 SNPs located in *MAP3K5*, thymocyte selection associated high mobility group box (*TOX*), nitric oxide synthase 1 (*NOS1*), nitric oxide synthase 2 (*NOS2*), arginase protein 2 (*ARG2*), and vascular endothelial growth factor receptor 1 (*FLT*) genes, where in the latter case a particularly high correlation with HU response was shown. The above results have also shown a strong correlation with the disease severity [68]. A few years later, during an independent validation cohort study, Koliopoulou and co-workers assessed the importance of the aforementioned genes (*N* = 5) and SNPs (*N* = 17). In this study, 87 participants of Greek descent were recruited with different phenotype severity (from moderate to severe) and interestingly, the majority of results remained statistically significant. According to the authors, the rs9376230, rs944725, and rs10483801 variants located in the *MAP3K5*, *NOS2A*, *ARG2* genes were considered as potential pharmacogenomic biomarkers, and subsequently, *FLT1* (rs2182008) and *ARG2* (rs10483801) genes were significantly associated with HbF levels and disease severity, respectively [69]. Furthermore, two independent research studies strongly suggest that the rs3191333 tag-SNP in the 3′-UTR of the *KLF10* gene is involved in HbF production and must be assessed as a discrimination marker between responders and non-responders to HU treatment, on β-type hemoglobinopathies [70, 71]. Notably, in the first study, a whole transcriptome analysis was performed in patients with β-thalassemia/SCD compound heterozygotes, with Hellenic origin (*N* = 25) who were treated with HU. Apart from the significant importance with HU efficacy, the rs3191333 tag-SNP could potentially be distinguished as a genetic biomarker in terms for β-thalassemia severity [70]. Subsequently, Elfalfy and co-workers were also validated the significant correlation of the mentioned Tag-SNP with HU efficacy, by performing a genotyping analysis in 75 patients of Egyptian origin [71].

In view of the foregoing, the application of pharmacogenomics provides a wide range of possibilities. The fact that the abovementioned results were confirmed in different population groups and types of hemoglobinopathies underscores the importance of the findings. Nevertheless, big data sets are being evaluated and analyzed in-depth and hence are constantly being updated, owing to the newly developed technologies and dynamics of high throughput sequencing studies, such as phenome-wide association studies (PheWAS) or GWAS [72, 73]. This combinational methodology attempts to identify a plethora of genetic information such as pharmacogenomic traits, mapping of genes, and genotype-phenotype associations for future targets and drug development [74, 75]. Overall, harnessing the power of pharmacogenomics may usher in tailored drug therapy for β-type hemoglobinopathies patients. Given the current stage, the significance in a series of biomarkers is being evaluated; however, more in-depth studies are appraised as essential. A summary of all the possible pharmacogenomic biomarkers to HU treatment is presented in Table 1 [68, 77–89]. Apart from the cognitive enhancement as regards the drug response, pharmacogenomics has significantly contributed to the better understanding of disease forms and the molecular mechanisms in hemoglobin switching. In fact, some of these findings distinguished regulatory elements such as the erythroid transcription factor (GATA1), BCL11A, and transcription factor SOX 6 (SOX6), critical for HbF silencing, where have recently been used as editing targets in gene therapy [90–93]. The potential of using pharmacogenomics as genome-guided treatment is strengthened even more.

**Table 1:** List of the currently available studies unrevealing the correlation of specific genes and genomic variants with hydroxyurea treatment efficacy. Findings were obtained using search engines databases such as PubMed Central (PMC-NCBI), dbSNP [76], and based on findings from our previous work [25, 69]

Disease	***N*** of patients	Ancestry	Gene(s)	dbSNP rsID	Location	Reference
SCA	137	African American	*HAO2* *MAP3K5* *MAP3K5* *TOX* *TOX* *TOX* *TOX* *TOX* *NOS1* *NOS1* *FLT1* *FLT1* *FLT1* *ARG2* *ARG2* *NOS2A* *NOS2A*	rs10494225rs9376230rs9483947 rs826729 rs765587 rs9693712 rs172652 rs380620 rs816361 rs7977109 rs9319428 rs2182008 rs8002446 rs10483801 rs10483802 rs1137933 rs944725	Untranslated Intronic Intronic Intronic Intronic Intronic Intronic Intronic Intronic Intronic Intronic Intronic Intronic Intronic Intronic Synonymous Intronic	[68]
TDT, NTDT, Hb S/β-Thal	87	Hellenic	*MAP3K5* *NOS2A* *ARG2*	STR 5’-GCGCG-3’ rs944725 rs10483801	Promoter Intronic Intronic	[69]
β-thal major, Intermedia, Hb S/β-Thal	138	Hellenic	*MAP3K5* *MAP3K5*	rs9483947 rs9376230	Intronic Intronic	[88]
β-thal major, Intermedia, Hb S/β-Thal	143	Hellenic	*KLF10*	rs3191333	3´‑UTR	[70]
β-thal major, Intermedia, SCD	75	Egyptian	*KLF10*	rs3191333	3’-UTR	[71]
β-thal major, NTDT, Hb S/β-Thal	165	Hellenic	*SIN3A*	rs7166737	Intronic	[71]
β-thal major, NTDT, Hb S/β-Thal	210	Hellenic	*KLF4*	rs2236599	Non-coding transcript exon variant	[63]
β-thal major, Intermedia	79	Western Indian	*HBG2*	XmnI polymorphism	promoter	[83]
SCD	150	N/A	*HBG2*	XmnI polymorphism	promoter	[76]
β-thal major	45	N/A	*HBG2*	XmnI polymorphism	promoter	[77]
β-thal major	133	Iranian	*HBG2* *HBB*	XmnI polymorphism	promoter	[78]
β-thal major	143	N/A	*HBG2*	XmnI polymorphism	promoter	[79]
β-thal major	54	Algerian	*HBG2*	XmnI polymorphism	promoter	[80]
β-thal major	18	N/A	*HBG2*	XmnI polymorphism	promoter	[81]
β-thal intermedia	37	N/A	*HBG2*	XmnI polymorphism	promoter	[82]
β-thal major/intermedia	81	Iranian	*HBG2*	XmnI polymorphism	promoter	[85]

*N/A* Not applicable, *NTDT* Non-Transfusion dependent thalassemia, *TDT* Transfusion dependent thalassemia, *SCD* Sickle cell disease, *SCA* Sickle cell anemia, *Hb S/β-Thal* compound heterozygous condition

### Use of databases: a roadmap for β-type hemoglobinopathies

A better understanding of the pathophysiology and characterization of diseases is a prerequisite to proceed with invasive and therapeutic approaches. Current well-structured databases contain significant data and therefore constitute an integral part of future targeted therapies. Databases contain sources, such as observational cohort studies, patient studies, laboratory reports, and clinical trials available to the science community at a global level. These sources are also known as real-world data (RWD) [94]. A variety of disciplines use RWD to associate data sets (usually dissimilar) in different ways and therefore proceed through validation studies in order to discover new markers that could lead to patient’s stratification and targeted therapies [75]. Some of the most important databases for β-type hemoglobinopathies and how they contribute to therapeutics are described below.

*The Syllabus of Human Hemoglobin Variants* (1996, first edition and 1998, second edition) consists of the first organized database including all the known human hemoglobin variants for understanding the pathophysiology of hereditary blood disorders, up to date [95]. A plethora of information such as hemoglobin abnormalities, amino acid substitutions, and/or DNA sequence alterations, even the geographic and ethnic distribution of the variants, along with clinical data and reports, are provided. Later, in 1997, a database titled “A Syllabus of Thalassemia Mutations,” was published. It constitutes a database specialized in the understanding of hematological malignancies and abnormalities, including hemoglobinopathies [96].

One of the most important hemoglobin databases, nowadays, is HbVar [97]. It is a locus-specific database providing information on the numerous genomic variants leading to different forms of hemoglobin variants and hence in different types of thalassemia and hemoglobinopathies [98]. The records of the database include detailed information about the ethnic occurrence, mutation frequencies, biochemical and hematological effects, and extensive phenotypic descriptions. Moreover, HbVar contains information about the frequencies of variants causing β-type hemoglobinopathies in at-risk populations. According to the latest update of HbVar, more than 1300 naturally occurring hemoglobin variants have been identified, including a wide range of insignificant to severe variations [98, 99]. A great benefit of HbVar is the interconnection with other genetic databases, such as FINDbase [100, 101] and Leiden Open-Access Variation database [102, 103]. Characteristically, Giardine et co-workers (2021) using the aforementioned databases, in a data mining effort by implementing microattribution approach, unrevealed a significant number of unpublished variants and HbF inducer targets for β-type hemoglobinopathies patients [99, 104]. Important findings using RWD are consistently updated through HbVar unrevealing further insertion and different types of mutations, such as rare or silent mutations [105].

In addition, ClinVar [106] and OMIM [107] may serve as additional and useful databases, from which researchers can retrieve information either about variants contributing to susceptibility or risk against a certain disease or about variants (clinically relevant) which affect strongly the response to a certain drug treatment. In comparison with OMIM, ClinVar includes some extra features such as genomic variants that are reported to affect drug response [108, 109]. At last, the 1000 Genomes project database [110] can be an alternative option for β-type hemoglobinopathies, especially for the association of human genetic variations, among different populations. Compared to others, all the included reports have been exclusively implemented with the latest sequencing technologies [111]. It should be noted that all the above databases are freely accessible.

## Gene therapy for hemoglobinopathies

Over decades, gene therapy strategies were considered by the majority of the scientific community as the most promising therapeutic approach in a variety of diseases. Gene therapy is defined as an experimental method where a recombinant genetic material is inserted into cells through a carrier to correct or compensate an abnormality. Carriers are usually genetically engineered vectors, responsible to deliver the corrected gene and its expression within humans at certain levels, depending on the disease [112]. Gene therapy has been used with great success to a variety of immunodeficiencies, and as expected, its application has been widely extended to other chronic inherited diseases, including β-type hemoglobinopathies [113].

Autologous transplantation of genetically corrected hematopoietic stem cells (HSCs) in patients with SCD and/or β-thalassemia is appraised as a gold-standard method, with high-efficacy and low-transplant risks. HSCs are used as target cells due to a variety of unique beneficial properties such as long-term or lifelong self-renewal ability and multilineage differentiation. Gene therapy with HSCs is one of the most attractive treatment options utilizing gene addition or genome editing technologies that can be applied either in vivo or ex vivo within a patient, depending on the treatment [114, 115]. In principle, in vivo gene transfer is usually administered through intramuscular, intrabone, or intravenous infusion in order for the corrected gene to be inserted and be expressed systematically in the recipient organism (gene insertion technologies). An alternative approach concerns the ex vivo reprogramming and cell manufacturing of the HSCs (genome editing technologies) and thereafter autologous transplantation into the recipient organism [116]. Nevertheless, the transition point from cell manufacturing to transduction seems quite challenging and in most cases, "gene delivery systems" determine the outcome, especially for β-type hemoglobinopathies, due to their complexity. [117, 118].

### Gene delivery systems—vectors

**Fig. 1:**
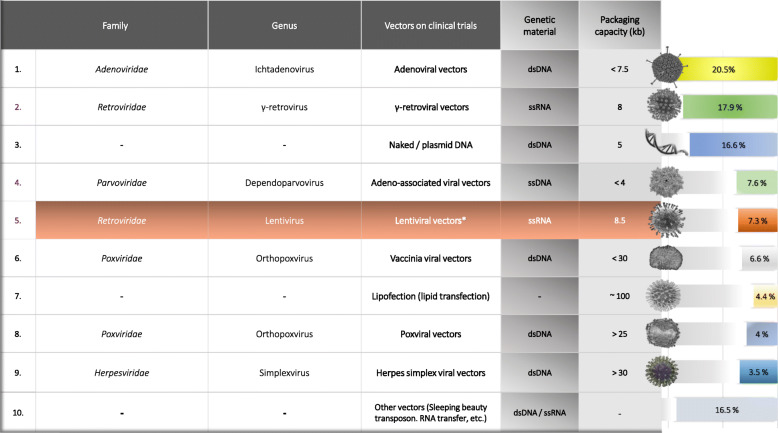
Comprehensive overview: viral and non-viral vectors in gene therapy clinical trials. Genus and family of viruses are reported, according to the International Committee on the taxonomy of viruses (ICTV) system. Information about the genetic material, approximate vector capacity (transgene insertion, etc.), morphology (3D structure) of each vector, are also included. On the chart (right side), the application percentage per vector in gene therapy clinical trials is displayed (based on Ginn and co-workers, 2018, John Wiley and Sons, Journal of Gene Medicine. 2018). Among them, vectors Nos. 2, 4, and 5 (colored purple) are also used in gene therapy clinical trials on β-hemoglobinopathies patients, and among them, lentiviral vectors are the most common (colored light orange row with an asterisk). The asterisk has been used to point out the significance of lentiviral vectors on β-type hemoglobinopathies and in addition, as these types of vectors are linked with Fig. 2, demonstrating their performance in clinical practice

During the design and selection of the proper vector, a variety of factors and features (e.g., genomic stability, cargo capacity, transduction efficiency) must be addressed in order for the transfection in the selected erythroid lineage to be accomplished successfully through an efficient and safe way to autologous HSCs [118, 119]. Recent findings and improvements on vectors have largely offset previous obstacles by leading the whole process of gene therapy to a new era. Indicatively, the major significance of vectors and their frequency in the clinical trials is presented in Fig. 1.

In general, vectors are classified into two types, viral and non-viral vectors. The first case includes recombinant viral particles and the second, non-viral vectors that can be either chemical (cationic lipids, peptide-based vectors, or solid lipid nanoparticles, etc.) or physical compounds (electroporation, magnetofection, hydroporation, etc.). Despite some important advantages of non-viral vectors, such as the large capacity and affordable manufacture, the low delivery efficiency during the transgene transfer and expression into the targeted cell lines is the major obstacle to its widespread application [120, 121]. Conversely, viral vectors seem to be a more reliable option, in most cases, due to their ability to transduce in vivo survival HSCs with a long-term transgene expression leading to the selection of viral vectors as a prerequisite [117].

Advances in gene delivery systems have significantly contributed to the upgrading of gene therapy by making the therapeutic approach of patients with inherited disorders feasible. Around β-type hemoglobinopathies, the most commonly used types of carriers include lentiviral and retroviral, adeno-associated vectors, and most recently specific non-viral vectors (sleeping beauty vector) that have been already proceeded in next-generation clinical trials [122]. Each type of vector is selected and reconstructed depending on the circumstance and characteristics of the disease. In the following paragraphs, we present a brief historical overview about vectors and technologies, used or being developed in β-thalassemia and sickle cell disease, with the aim of better understanding the background underlying gene therapy.

## Viral vectors

### Retroviral vectors

In the beginning, early-phase trials launched using classical Moloney murine leukemia virus (MLV)-based retroviral vectors which have been used widely to carry corrected genes (β-globin gene) to transduce HSCs, in murine models [123–126]. Retroviral vectors have been used for over 20 years for in vivo and ex vivo gene therapies with significant advantages such as the long-term and stable transgene expression into the host genome, owing to their ability to infect only dividing cells (hematopoietic stem cells). On the other hand, the permanent integration of the vector genome into the host genome can pose additional risks and, in particular, there is a possibility of triggering cellular oncogenesis, as the transgenic material is randomly integrated, which can lead to the development of malignancies [115, 127, 128]. Later, the development of self-inactivating retroviral vectors was a major step forward to reduce the aforementioned risks, at least in theory; however, the integration mechanisms of retroviruses remain quite where they usually lead to sub-therapeutic levels, in terms of efficiency. In some cases, even if the gene transfer has has successfully been applied in mouse repopulating stem/progenitor cells, findings have shown that the expression of the β-globin gene cannot reach the required therapeutic levels [129]. A new set of eliminations and additions of important elements, necessary for β-globin, such as (intron modifications, the addition of β-LCR, etc.) had a positive impact but was still lacking transduction efficiency and β-globin gene expression in HSCs. In summary, retroviral vectors can be considered as a viable option for gene therapy due to recent advances but further improvement and investigation are deemed necessary [130].

### Lentiviral vectors

A few years later, to overcome the aforementioned issues, new reconstructive lentiviral vectors were developed. These types of vectors are based on human immunodeficiency virus type 1 (HIV-1) and unlike retroviral, they acquire significant advantages such as the ability to transduce nondividing cells with relative genomic stability to accommodate both full LCR and adult β-globin gene, containing the transcribed genes-targets and DNA fragments. Furthermore, the majority of preclinical and clinical trials have shown that the possibility of developing mutagenesis is significantly lower than in retroviral vectors. In terms of safety and efficiency, the construction of self-inactivating lentiviral vectors (SIN) allows the viral long terminal repeats to be removed upon integration and at the same time increases the cargo capacity, including all the necessary regulatory elements and the targeted gene, to succeed a higher transgene expression [131]. In 2000, a pioneering study described SIN lentiviral vectors carrying the human β-globin gene to be able to transduce genetically corrected HSCs in a safe and efficient manner, in murine models with major β-thalassemia [132]. Similar results were documented in independent studies, even using other transgenes such as the γ-globin gene, indicating that lentiviral vectors (LVs) can be transferred to advanced trials. In the following years, these findings were followed by continuous upgrades in both ex vivo and in vivo studies, leading to further research. Recently, the TNS9, sGbG, HPV569, BB305, and GLOBE vectors have been mentioned as “great options” for gene therapy [50, 115, 118, 133, 134]. Using an ex-vivo approach and different sets of LVs, pediatric patients with β-thalassemia were also treated in other clinical trials [134–136]. In the case of SCD, despite the efficient transduction, the expression of an effective anti-sickling β-globin gene sometimes was missing [137], being LVs efficiency under discussion. Nevertheless, clinical trials using lentiviral vectors have also been launched and in comparison to β-thalassemia are presenting low success rates. It is important to mention that recent advances and additions of specific agents consist a ray of hope for SCD [138, 139]. Overall, lentiviral vectors are the most commonly used gene delivery tools in patients with β-type hemoglobinopathies.

### Adeno-associated viral vectors

Besides the retroviral and lentiviral vectors, other alternatives viruses have intensively gained attention as potentially useful vectors on β-type hemoglobinopathies. Adeno-associated virus is a non-pathogenic human parvovirus with low immunogenicity and cytotoxicity and due to a series of advantages has extensively been studied for gene therapy. In particular, adeno-associated vectors (AAVs) cause a very mild immune response in humans and are able to infect quiescent and non-dividing cells with a broad host and cell type tropism range [121, 140, 141]. Last but not least, it has been reported that gene expression can be maintained at high levels for a long-term period. Concerning β-type hemoglobinopathies, to both in vitro and in vivo studies, more often are used specialized recombinant AAV vectors (rAAVs) or self-complementary (scAAVs) in order to overcome some major limitations in comparison to the typical ones, such as the limited cargo capacity and the slow onset of gene expression [142, 143]. Notably, successful transductions in both murine and human HSCs using recombinant rAAVs vectors have shown great potentials and especially in the case of the AAV2 and AAV6 serotypes [144–146]. Recently, Yang and co-workers (2020) suggested that AAV6, under certain circumstances, can be used for gene therapy in β-type hemoglobinopathies [147]. Of course, additional future research studies for the optimization of these types of vectors to avoid some weaknesses will be beneficial in the future. Indicatively, the AVV vectors, despite the improvements, are capable to transfer a relatively small transgene. Furthermore, the integration of the transgene into the genome randomly should be considered. Despite the improvements, AVVs vectors and most recently in combination with genome editing technologies hold the promise to be extensively used as alternative vectors in gene therapy [122, 148, 149].

## Non-viral vectors

A slightly different gene delivery technology can be provided through the use of non-viral vectors; DNA plasmids can be transduced even as “naked DNA” or with different supportive compounds into the targeted cells. A combination of features, such as the large cargo capacity, avoidance of the immune system, and overall safety of use as an intervention tool, and of course, the low cost, make the non-viral vectors an attractive option [150]. Nevertheless, there are still important limitations that need to be overcome in order to be used widely on a clinical trial level (approximately 30% are ongoing within clinical trials on gene therapy), as they possess low transgene expression levels, transduction efficiency and stability [151].

For many years, it was considered an alternative option under development. However, after some recent crucial reconstructions (improved versions of the transposase “Sbase,” and integration cassette “transposon”), non-viral vectors and in particular, a hyperactive transposon system called the Sleeping Beauty (SB) have shown great results, in disease models [151, 152]. In 2009, two independent studies by using different versions of SB transposon systems have successfully delivered genetic modifications in the primitive human cluster of differentiation 34+ (CD34^+^) HSCs, with stable transgene expression [153]. According to the latest updates, the clinical potential of SB vectors for patients with SCD has been also highlighted. Different applications and research studies in disease models are currently in the preclinical phase and even different alternative stem cell sources (iPS, for instance) are under investigation as quite promising alternative approaches [154, 155].

Moreover, during the last few years, a new generation of technology (CRISPR/Cas9 system) in combination with transposon-based vectors such as PiggyBac gained momentum, showing some early phase studies their efficient applicability in a variety of diseases [156]. For example, Xie et co-workers (2014) using PiggyBac combined with the CRISPR/CAS9 system managed to correct an abnormal *HBB* gene, including point variants, in iPS cells, for β-thalassemia. Based on the results, PB-CRISPR/Cas9 platform provided a stable *HBB* gene expression in iPS-derived erythroblasts upon hematopoietic differentiation [157].

All the above underscores the progress that has been made regarding the proper use and development of vectors in gene therapy for β-type hemoglobinopathies. Viral vectors have been significantly improved in terms of safety, efficacy, and financial viability. The new generation of vectors provides a more stable and efficient, at high-levels, transgene transfer and delivery into the host cell as well as low immunogenicity and genotoxicity. Also, the high proportion of integrations into specific sites (genomic safe harbors, GSHs) ensures that the newly inserted genetic elements will be functional and moreover any alterations of the host genome will be avoided [158]. From the aspect of financial viability, the cost of viral vectors has been adjusted to serve a larger number of patients in different types of diseases and therefore, among other reasons, are highly preferred. As for non-viral vectors, recent improvements, given the low cost, indicate that it may be a viable alternative in the future.

## Clinical applications in patients with β-type hemoglobinopathies

### Gene addition technologies

Briefly, gene addition technologies refer to a one-time treatment gene therapy approach. This process can be divided into 3 phases including the collection of patient’s stem cells, ex vivo cell manufacturing, where a transgene is inserted into the stem cells through modified vectors, a process called transduction, and thereafter genetically corrected HSPCs are inserted into the recipient patient, aiming to deal with β-hemoglobinopathies. During ex vivo gene therapy, chemotherapy and conditioning agents are important key-stages for a successful treatment. As noted, autologous transplantation can offer higher rates of success and a series of benefits in comparison with allogeneic transplantation, and moreover, some obstacles such as histocompatibility complex and transduced risks can be overcome [114, 159]. For decades, continuous efforts have been made to optimize ex vivo gene therapy, with unsolicited results. A series of recent successful trials using autologous transplantation of genetically corrected HSCs in combination with specialized lentiviral vectors, in murine models, have made clinical trials into humans acceptable [160]. Numerous findings have confirmed that in terms of efficacy and safety, gene therapy is carrying low-transplant-related risks, providing a long-term repopulation of corrected HSCs, and could be available to a wide range of patients with β-thalassemia and/or SCD [135, 161, 162]. In 2013, clinical studies of investigational gene therapies were approved in patients with severe sickle cell disease and β-thalassemia, under the auspice of Bluebird bio and others. These include the St. Jude Children’s Research Hospital, Cincinnati Children’s Hospital Medical Center, University of California, Memorial Sloan Kettering Cancer (United States), and IRCCS, San Raffaele (Italy), and Bluebird Bio (United States, Thailand, Australia, France) [120, 163]. In The last 5 years, several successful cases have been recorded using gene addition technologies. The most recent findings regarding clinical trials are described below.

### β-thalassemia

In 2007, two patients with β-thalassemia and one patient with transfusion-dependent HbE/β-thal were successfully treated using gene therapy. These patients were transduced with HPV569 lentiviral vector, carrying β^Τ87Q^ globin, and remarkably in the case of the patient with transfusion-dependent HbE/β-thalassemia his phenotype improved by becoming transfusion independent with a gradual and significant increase in gene-marked cells up to 10–20% and stable Hb levels. The maintenance of HbF therapeutic levels and clonal persistence lasted about 9 years [135]. This pilot study is considered as milestone for the clinical development of gene therapy and has led to a number of clinical trials in patients with β-type hemoglobinopathies [112, 164]. A few years later, two subsequent clinical trials were launched (2013), in patients with transfusion-dependent β-thalassemia (*N* = 13) resulting in significant benefits. In particular, using the BB305 vector that expresses βT87Q globin, the majority of patients reduced or even eliminated the clinical severity of the disease. Based on the latest report for active gene therapy clinical trials, 35 out of 50 transfusion-dependent thalassemia patients, between ages 5 and 64 years old, were successfully treated, and in most patients, the transfusion was reduced or discontinued. The majority of the patients did not show any severe adverse events in relation to vector integration and gene therapy conditions [128, 164]. In 2018, among the aforementioned patients, a new protocol was designed where the transduction has been made in CD34^+^ cells, using a new vector called GLOBE, and then administered by intraosseous infusion to the posterior-superior iliac crests. Remarkably, 9 out of 10 patients with different genotypes were successfully treated, with no evidence of transduction-related risks and abnormalities or adverse events [130, 138].

Summarizing, the application of gene therapy has significantly improved in patients with transfusion-dependent β-thalassemia over time. Some of the most important upgrades in the gene therapy process are included the design of the new GLOBE vector, the combined use of plerixafor and granulocyte colony-stimulating factor (G-CSF) for the mobilization of HSPCs and in addition, the replacement of busulfan with treosulfan and thiotepa-based conditioning, as myeloablative regimens [138]. However, in patients with severe symptoms and in particular in cases with major β-thalassemia, gene therapy application can be denoted as a more complicated and less effective procedure.

### Sickle cell disease

Gene addition technologies were also used in patients with SCD. The first clinical development of gene therapy was done in France (NCT02151526) in a patient with SCD. This patient was treated with autologous transplantation of lentiviral-corrected hematopoietic stem cells (with the BB305 lentiviral vector, expressing the β^A-T87Q^ globin [162]. Despite this first successful attempt, upcoming clinical trials did not represent similar results, as anticipated. One of the major obstacles was the harvesting and immunoselection of patients’ stem cells from the bone marrow. An inability of transduced HSCs to get robustly mobilized in the peripheral circulation has been reported in a considerable number of patients. Filgrastim, one of the most common mobilizing agents for SCD, despite its high efficacy to mobilize large numbers of hematopoietic stem cells, is not recommended in terms of safety as serious adverse events have been reported in patients with SCD [160, 162]. However, the recent replacement with plerixafor, an alternative HSC mobilizer, has improved the efficiency of mobilization and isolation of both hematopoietic stem and progenitor cells, and with great interest, no adverse events were observed [165]. Similar findings of Fritolli’s and co-workers (2011), in patients with transfusion-dependent β-thalassemia patients, enhanced once more the evidence that plerixafor causes higher mobilization of HSCPs from the bone marrow into the peripheral circulation [166]. After several subsequent studies and trials, plerixafor is now used in ongoing active gene therapy clinical trials. Based on the latest report, (sponsored by Bluebird Bio and others), 14 out of 59 patients have successfully been treated. Apart from mobilization agents, in addition of new specialized vectors such as bAS3-FB, mLARbDcV5, LCR-shRNA^mir^, and cell manufacturing conditions resulted in an increment in the rate. of success. Nevertheless, clarification of the network of HSCs in the bone marrow microenvironment along with further improvements in cell quality and dose will improve the transduction efficiency and hence the overall application of gene therapy in SCD [130, 138, 164].

**Fig. 2:**
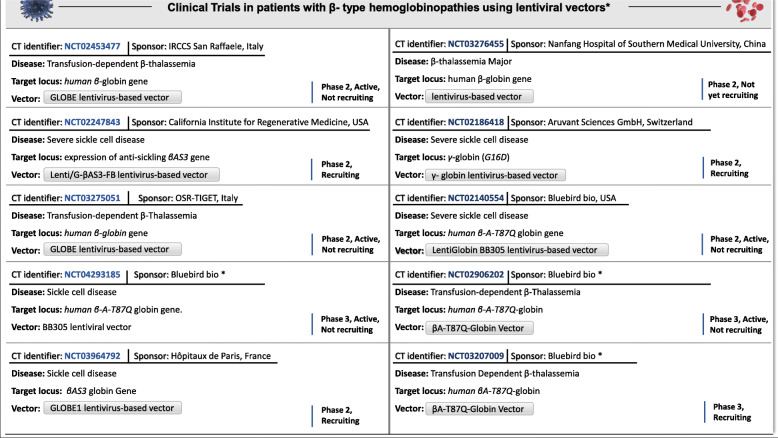
Clinical Trials in β-hemoglobinopathies patients using lentiviral vectors (source of data: [76] Assessed 13-11-2020). The total number of results was performed using certain filtering criteria: “Recruitment status: Not yet recruiting OR Recruiting OR Active, not Recruiting,” “Sex status: All,” “Study type: Interventional (Clinical Trial),” Study Phase: Phase 2 OR Phase 3, Period of clinical trial: 2010 to 2020. The asterisk in clinical trials identities: NCT04293185, NCT03207009, NCT02906202 symbolizes that these clinical trials are sponsored by Bluebird Bio and have been accepted to phase 3. In this phase, the recruitment includes an increased number of participants, in different locations and populations.

In summary, gene addition technologies are constantly being upgraded and could potentially be integrated into the clinical routine, in the near future. Among others, lentiviral vectors possess a leading role in current clinical trials of patients with β-types hemoglobinopathies and so far, the results are quite optimistic (see Fig. 2). However, there are some obstacles as regards the difficulty level, expertise and procedure complexity. Some of the critical issues and limitations include the pre-analysis (cell manufacturing) and analysis phases such as the proper source selection of the expansion. Moreover, important factors such as eligibility criteria, route of administration, dose, and quality of the cells need to be further taken into consideration [138, 161]. For that reason, other clinical applications such as genome editing technologies are in progress using different approaches and methodology, aiming to overcome the aforementioned issues.

### Genome editing technologies

Nowadays, genome editing approaches have gained great momentum in health sciences and despite recent emergence, they have monopolized interest in the scientific community as a possible means of treating or preventing a variety of diseases. As mentioned, in gene addition technologies, the genetic material is delivered into the genome, randomly, whereas genome editing technologies are able to intervene with an organism’s DNA and genetic material can be added, removed, or altered on a specific genomic location, through a precise and permanent way [148]. High accuracy rates are observed due to the specialized identification and processing systems that these technologies possess [167]. To a large extent, the proper selection of nuclease will define whether double-strand breaks (DSBs) will identify the targeted-precise locations into the genome, allowing the gene editing. The most common and efficient genome editing systems include the zinc-finger nucleases (ZFNs), transcription activator-like effector nucleases (TALENs), or clustered regularly interspaced short palindromic repeats/CRISPR-associated protein 9 (CRISPR/Cas9) [148, 168, 169]. From all of them, CRISPR/Cas9 system has shown to be the most efficient and applicable method, until today.

Various genome editing strategies have been successfully devised to treat different maladies, including β-type hemoglobinopathies. Most of the pre-clinical studies have been focused on investigating genes, transcription factors and variants that are involved in the switching from HbF to HbA haemoglobin or to HbF inducer drugs. [170, 171]. Researchers have exploited a set of this information and according to the following findings, a series of therapeutic interventions using editing approaches have successfully been implemented in mouse and human models for both SCD and β-thalassemia. These independent studies involve SOX6, BCL1AA, and KLF1 transcription factors and others (e.g., activators, repressors) with different genome editing technologies (CRISPR/Cas, ZNFs systems, etc.) and strategies (gene knock-in, knockdown or knockout). These approaches have led to the desired globin expression and therefore to reactivation of fetal hemoglobin, and in some cases to the correction of the α/ß globin chains imbalance resulting in phenotype improvement [91, 172–174]. In addition, through the use of CRISPR/Cas9 structural mutations within specific promoters in the hemoglobin subunit gamma-1 (HBG1) and hemoglobin subunit gamma-2 (HBG2) genes have been successfully corrected, demonstrating some of the significant benefits of those systems and how they can be harnessed [175–178].

As previously mentioned, major upgrades in vectors combined with genome editing technologies and especially the CRISPR editing system, have over boosted genetic engineering in terms of efficacy and transgene transduction. A representative example concerns the correction of the ß-globin gene variants using TALEN, ZFNs, or CRISPR/Cas9 nucleases combined with single-strand oligodeoxynucleotide donors, integrase-defective LVs, or rAAV6 carrying the donor templates in SCD HSCs cells [179–182]. Applying these systems, DSBs are created at the mutation surrounding the DNA sequence, which then get corrected using a delivered normal copy of the ß-globin gene. This site-specific correction of the sickle mutation in HSCs also allows the permanent production of normal red blood cells [139, 181].

Over the last years, different genome editing strategies were tested for SCD and thalassemia, using both in vitro and/or animal models. Genome editing technologies in β-type hemoglobinopathies until recently were only being used for ex vivo gene editing aiming to correct the β-globin gene mutations or the induction of endogenous fetal-globin. [93, 172, 178]. All the above findings and a number of others led genome editing technologies from the bench to clinical trials in order to be used in autologous gene therapy as a principal genome-based therapeutic intervention. Specifically, an investigational therapy called “CTX001” was launched for patients suffering from β-thalassemia and SCD in which a series of clinical trials are ongoing [183]. In the clinical trial NCT03728322, HSCs from patients with β-thalassemia will be edited by impairing the abnormality, and thereafter HSCs will be re-inserted into the patient. Other promising future clinical trials include patients suffering from β-thalassemia (NCT03655678, NCT03432364, NCT04208529) and SCD (NCT03745287, NCT03653247, NCT04208529) in which hematopoietic stem cells are engineered using ZFNs or CRISPR-Cas9 to produce high levels of fetal hemoglobin in red blood cells, through *BCL11A* disruption [149, 174, 184]. To date, the upcoming results from the CTX001 project have shown great promise to be used as a future strategy. In 2019, the first patient with SCD was treated by gene-editing CRISPR-Cas9 technology (CTX001) as part of a phase 1 trial in the USA. Furthermore, BCL11A targeted disruption using the ZNFs system was also achieved in human bone marrow stem cells, with the upregulation of fetal globin expression in erythroid cells [185]. The elevation of fetal hemoglobin can alleviate transfusion-requirements for β-thalassemia patients and painful and debilitating crises for SCD patients.

Overall, it is important to highlight that the new clinical genome editing approaches for hemoglobinopathies still need to be evaluated in clinical efficacy, genotoxicity, safety studies, and other limitations or issues issues which may be caused by the application of these technologies. Since CRISPR technology is expected to be highly used in the clinical setting, computational and experimental tools were or are being developed to predict these events. Concerning some disadvantages, in vivo gene therapy can lead to secondary effects due to off-target genome editing, inefficient or off-target delivery, and stimulation of autoimmune responses. Even the CTX001 technology that is already included in human clinical trials necessitates careful monitoring and observation [183, 186]. Generation of hyper accurate CRISPR systems are being assessed as a future strategy by altering the Cas9 protein or even its substitution by proteins like centromere and promoter factor 1 (CpfI), Cas12, or 13 to reduce off-targets [187, 188]. Besides, comparative studies and targeting analysis with the already implemented treatments should establish advantages or disadvantages in terms of potential therapeutic benefit versus the current future studies [189]. Nonetheless, genome editing therapy holds real promise for patients with SCD and β-thalassemia and it can potentially improve patients' survival rates as well as the quality of life by making autologous gene therapy acceptable.

## Future perspectives

Genome-based therapeutic interventions have a notable role in both drug and gene therapy. The application of genome-guided treatment is a rapidly evolving strategy with many possibilities, but still, the development of pharmacogenomic testing is in the early-phase investigations for a variety of reasons. First of all, there are a limited number of approved drugs for β-thal and SCD, and thus pharmacogenomics cannot be exploited. It is estimated that in the future multiple drugs will be developed as the majority are at an advanced stage [22, 190]. An additional obstacle for drug treatment refers to the high level of difficulty that arises when molecular diversity of β-type hemoglobinopathies results in a series of barriers to detection of novel biomarkers, across different groups of patients and populations. Next-generation sequencing technologies have shown great potentials in genomic research by expanding our knowledge among genomic variations, disease development, or treatment response and thus we expect to obtain a better stratification of all the above soon [191, 192]. Last, but not least, genome editing technologies, particularly CRISPR, can be utilized through different ways and directions either to reveal and validate novel drug targets or to explore effects on drug activity, based on the genetic makeup. In addition, CRISPR is quietly revolutionizing the search for new drugs, transforming drug discovery into a new era, and hence it might be instrumental to inherited disorders [186].

On the other hand, gene therapy as a direct genome-based therapeutic intervention seems to be going through a golden age in β-type hemoglobinopathies and recently recently updated integrated tools and technologies have led its application one step forward into clinical translation. In the last 5 years, a new generation of technologies and delivery tools have made autologous transplantation of genetically corrected HSCs feasible. For this to happen, cell manufacturing, proper selection of vectors, and efficient transgene transduction as the long-term expression need to be adapted and improved depending on each case and disease [148, 159, 193]. The design of new reconstructed viral and non-viral vectors combined with genome editing methods have shown important clinical benefits but in certain types of β-hemoglobinopathies and circumstances [120, 194]. In terms of genome editing, in a short period of time, it has managed to revolutionize genome intervention methodologies, a valuable tool with wide application in health sciences but efficacy and safety need to be further assessed.

Beyond the existing strategies, steady improvement of our knowledge concerning the complex and highly heterogeneous molecular mechanisms governing hemoglobinopathies allows us to explore potential targets for future therapeutic strategies. Initially, to reach that level, improvements need to be made for the prevention and management of β-type hemoglobinopathies. As it is previously mentioned, identification of disease severity is a prerequisite for treatment selection. Current techniques and screening methods for the diagnosis of hemoglobin disorders usually fail to detect pathogenic deletions, point mutations, variants, etc. causing problems in the accurate diagnosis. In comparison with traditional methods, molecular diagnosis using the next-generation sequencing (NGS) technique may be able to overcome these obstacles [191]. In fact, Shang and co-workers (2017) designed a rapid targeted NGS platform for molecular screening and clinical genotyping which is capable to identify pathogenic or likely pathogenic variants [195]. The prevalence of sickle cell disease and β-thalassemia in low-outcome countries gives rise to even bigger problems in controlling its spread. Since the cost of the NGS has become more accessible and affordable, it might be a substantial solution for β-type hemoglobinopathies [196].

Finally, the economic evaluation and analysis of the above strategies will be in a position to determine whether these strategies can be adapted in healthcare systems. The spread of hemoglobinopathies has a worldwide long-term impact by weakening national healthcare systems and at the same time burdening annual health expenditures. Following our suggested therapeutic strategies, the cost varies considerably, depending on the applying technology, and opinions differ [197, 198]. Specifically, in autologous gene therapy or even when gene addition or genome editing technologies were used, the whole procedure is extremely expensive and therefore significantly complicates its widespread implementation [138]. Although, from another point of view, it is supported that in comparison with other therapeutic approaches, is qualified as a one-time, life-saving treatment, avoiding any any additional direct and indirect costs (hospitalization, supportive treatment). Drug therapy with hydroxyurea and the use of pharmacogenomics as indirect genome-based therapeutic intervention seems a more realistic scenario [199], even using next-generation sequencing technologies where the cost is estimated to be more expensive, compared to traditional genotyping methods. Additionally, both direct and indirect costs will be reduced as concern the need for hospitalization and blood transfusion [200, 201]. It is considered necessary for all of the above to be further assessed by performing a costing analysis (cost-effective, cost-utility, and cost-benefit analysis), in the near future [202].

## Conclusion

In summary, hemoglobinopathies are one of the world’s major health problems and since there is no definitive treatment, it remains quite a challenge. To the best of our knowledge, treatment in patients with β-type hemoglobinopathies  should not be a an “one size fits all” approach, especially in severe cases, as any attempt of widespread use of supportive treatments and common strategies has failed. For that reason, the idea of incorporating a more personalized approach into existing strategies or to the new ones has gained significant ground in the field of biomedical research. To date, both indirect and direct genome-based therapies have been adapted to different treatment strategies, sharing a common goal of finding alternatives for β-type hemoglobinopathies. These strategies are aiming either to provide a better quality of life or ideally to lead to permanent cure. On the one hand, personalized and targeted therapy with hydroxyurea has shown great benefits in the early phase process and is strongly suggested that upcoming technological advancements will further enhance its importance. On the other hand, significant advances in the whole process of in vivo and ex vivo autologous gene therapy raise the hopes to enter clinical practice. These upgrades mainly concern cell manufacturing, the design of a new generation of vectors where transduction efficiency is improved, and the use of alternative conditioning as well as mobilizing agents, achieving a long-term expression of corrected HSCs. In conclusion, genome-based intervention, in one way or another, can be used in a variety of ways, even in different strategies, suggesting their importance and their enormous potential to turn myths of the past into reality in the future. However, as already mentioned, there are still important parameters (biological, bioethical, and financial) that need to be assessed and further improved, and last but not least, to gain a deeper understanding of how these technologies can be implemented.

## Data Availability

Not applicable.

## Abbreviations

AAV6: Adeno-associated virus type 6
ARG2: Arginase protein 2
BCL11A: B cell lymphoma/leukemia 11A
β-thal: β-thalassemia
CD34^+^: Cluster of differentiation 34+
CpfI: Centromere and promoter factor 1
CRISPR/Cas9: Clustered regularly interspaced short palindromic repeats/CRISPR associated protein 9
DNA: Deoxyribonucleic acid
DSBs: Double strand breaks
EMA: European Medicines Agency
FDA: Food and Drug Administration (USA)
FLT: Vascular endothelial growth factor receptor 1
GATA1: Erythroid transcription factor
G-CSF: Granulocyte colony-stimulating factor
GSHs: Genomic Safe Harbors
GWAS: Genome-wide association studies
HB: Hemoglobin
HBB: Hemoglobin subunit beta
*HBG1*: Hemoglobin subunit gamma-1
HBG2: Hemoglobin subunit gamma-2
HBS: Sickle hemoglobin
HDACi: Histone deacetylase inhibitors
HIV-1: Human immunodeficiency virus type 1
HSCs: Hematopoietic stem cells
HSCT: Hematopoietic stem cell transplantation
HU: Hydroxyurea
IMiDs: Immunomodulatory imide drugs
iPS: Induced pluripotent stem cells
LV: Lentiviral vector
MAP3K: Mitogen-activated protein kinase 3
MLV: Moloney murine leukemia virus
mTOR: Mechanistic target of rapamycin
NGS: Next-generation sequencing
NOS1: Nitric oxide synthase 1
NOS2: Nitric oxide synthase 2
PB: PiggyBac, transposon-based vector
PheWAS: Phenome-wide association studies
rAAVs: Recombinant AAV vectors
RWD: Real-world data
SB: Sleeping Beauty transposon system
SCD: Sickle cell disease
scAAVs: Self-complementary vectors
shRNA: Short hairpin RNA
SIN: Self-inactivating vector
SNP: Single nucleotide substitution
SOX6: Transcription factor SOX 6
TALENs: Transcription activator-like effector nucleases
TOX: Thymocyte selection associated high mobility group box
USA: United States of America
ZFNs: Zinc-finger nucleases
α2β2; HbA: Adult hemoglobin
α2γ2; HbF: Fetal hemoglobin
β-LCR: Locus control region β
